# Influence of COVID-19 pandemic on the virus spectrum in children with respiratory infection in Xuzhou, China: a long-term active surveillance study from 2015 to 2021

**DOI:** 10.1186/s12879-023-08247-3

**Published:** 2023-07-13

**Authors:** Rundong Cao, Yangguang Du, Jing Tong, Dong Xia, Qinqin Song, Zhiqiang Xia, Mi Liu, Haijun Du, Jun Han, Chen Gao

**Affiliations:** 1 Center for Viral Resource, Chinese Center for Disease Control and Prevention, National Institute for Viral Disease Control and Prevention, Beijing, 102206 China; 2 Xuzhou Center for Disease Control and Prevention, Xuzhou, 221002 China

**Keywords:** Children, Respiratory virus, Coronavirus disease 2019, Prevention and control measures

## Abstract

**Background:**

To investigate the impact of the coronavirus disease 2019 (COVID-19) outbreak on the prevalence of respiratory viruses among pediatric patients with acute respiratory infections in Xuzhou from 2015–2021.

**Methods:**

Severe acute respiratory infection (SARI) cases in hospitalized children were collected from 2015–2021 in Xuzhou, China. Influenza virus(IFV), respiratory syncytial virus (RSV), human parainfluenza virus type 3(hPIV-3), human rhinovirus (hRV), human adenovirus(hAdV), human coronavirus(hCoV) were detected by real-time fluorescence polymerase chain reaction(RT-qPCR), and the results were statistically analyzed by SPSS 23.0 software.

**Results:**

A total of 1663 samples with SARI were collected from 2015–2021, with a male-to-female ratio of 1.67:1 and a total virus detection rate of 38.5% (641/1663). The total detection rate of respiratory viruses decreased from 46.2% (2015–2019) to 36% (2020–2021) under the control measures for COVID-19 (*P* < *0.01*). The three viruses with the highest detection rates changed from hRV, RSV, and hPIV-3 to hRV, RSV, and hCoV. The epidemic trend of hPIV-3 and hAdV was upside down before and after control measures(*P* < *0.01*); however, the epidemic trend of RV and RSV had not changed from 2015 to 2021(*P* > *0.05*). After the control measures, the detection rate of hPIV-3 decreased in all age groups, and the detection rate of hCoV increased in all except the 1 ~ 3 years old group.

**Conclusions:**

Implementing control measures for COVID-19 outbreak curbed the spread of respiratory viruses among children as a whole. However, the epidemic of RV and RSV was not affected by the COVID-19 control policy.

## Introduction

Since its outbreak in late 2019, the coronavirus disease 2019(COVID-19) outbreak has rapidly spread to many countries and regions worldwide, causing a global pandemic with an unprecedented and far-reaching impact on society [1, 2]. To curb the spread of the disease, China has implemented a series of effective public health precautions since the end of January 2020 [3], followed by a series of related precautions in many countries [4]. Studies show that these measures not only effectively curb the spread of COVID-19 [5, 6], but also affect the reach of many respiratory pathogens [7–10], such as causing an early end to the spread of Influenza virus(IFV) in many countries in the Northern Hemisphere [11, 12]. The epidemic season of respiratory syncytial virus (RSV) also changed significantly after the COVID-19 epidemic, and the detection rate of RSV decreased sharply all over the world [13]. However, Australian researchers find that the human rhinovirus (hRV) detection rate is higher than the previous average level since May 2020, which is quite different from the decreasing trend of most respiratory viruses [14]. Thus, the epidemiological trend of respiratory virus changes aroused our concern.

To explore the changes in the common respiratory virus spectrum before and after the outbreak, Xuzhou was selected as the monitoring target to observe the changes in viruses in China. Xuzhou City, Jiangsu Province, is an important city at the junction of the north and south of China, also a national transportation hub. Therefore, Xuzhou has a unique climate as well as population distribution characteristics. This retrospective study analyzed respiratory viruses in clinical samples from severe acute respiratory infection(SARI) children’s cases in Xuzhou, Jiangsu Province, from January 2015 to December 2021. We further analyzed changes in the respiratory virus spectrum before and after the COVID-19 outbreak to help improve preventive measures for viral infections in children.

## Materials and methods

### Clinical specimens

This study relied on a provincial sentinel hospital for acute respiratory infectious disease surveillance in Xuzhou City, Jiangsu Province. Case pharyngeal swab specimens and corresponding clinical questionnaires were collected from children with SARI hospitalized in Xuzhou Children's Hospital from January 2015 to December 2021.

### Research methods

- 1. Case definition: We used the 2011 WHO case definition for SARI for children > 5 years [15]: sudden onset of fever of > 38ºC and cough or sore throat in the absence of another diagnosis, shortness of breath or difficulty in breathing and requiring hospital admission; for patients < 5 years: cough or difficulty in breathing and breathing faster than 40 breaths/min (ages 1–5 years) or breathing faster than 50 breaths/min (ages 2–12 months); cough or difficult breathing and unable to drink or breastfeed, or vomits everything, or convulsions, or lethargic or unconscious, or chest indrawing or stridor in a calm child and requires hospital admission.
- 2. Sample collection: On the day of hospitalization or the next day, one pharyngeal swab was collected and stored at 4 °C. The swab was sent to the Xuzhou Center for Disease Control and Prevention within 72 h and stored in a refrigerator at -80 °C.
- 3. Nucleic acid extraction: The MagNA Pure 96 nucleic acid extraction apparatus (Roche, Switzerland) was used to extract the total nucleic acid from the specimens according to the operation manual of the instrument and the instructions of the kit. The total nucleic acid is stored at -80℃.
- 4. Reagents and nucleic acid testing: Detection of common respiratory viruses in specimens, including respiratory syncytial virus (RSV), human adenovirus (hAdV), human rhinovirus (hRV), Influenza virus A (IFV-A), Influenza virus B (IFV-B), human parainfluenza virus type 3 (hPIV-3), human coronavirus 229E (hCoV-229E), human coronavirus OC43 (hCoV-OC43). According to the sample size, the number of children positive for IFV-A and IFV-B was combined as IFV for analysis, and the number of children positive for hCoV-OC43 and hCoV-229E was combined as hCoV for analysis.

Primer probe sequences for all viruses are referenced in the literature [16]. Specimens were amplified and tested using the AgPath-IDTM One-step RT-PCR Kit kit (Thermofisher, USA) and a Roche LightCycler® 480 real-time quantitative fluorescence PCR instrument (Roche, Switzerland). Results were interpreted according to the manufacturer's instructions.

### Statistical analysis

Data quality control was performed using EpiData Version 3.1 for code and double-enter, and then imported to SPSS 23.0 for further analysis. Plotting was performed using Graphpad Prism 8.0. Count data were expressed as the number of cases and percentages, and the χ2 test was used to compare groups. *P* < 0.05 was considered a statistically significant difference.

## Results

### Total detection of respiratory viruses in children

From January 2015 to December 2021, a total 1663 SARI cases were collected, including 1040 male (62.5%) and 623 female (37.5%). The male to female ratio was 1.67:1. The median age of cases was 0.58 years (interquartile range [IQR]: 0.17–2 years). Among them, 982 cases were younger than 1 year old, 26 cases were 1 ~ 3 years old, 233 cases were 3 ~ 6 years old, and 122 cases were 6 ~ 18 years old. The detection of viruses was no significant difference in terms of gender. (*P* > 0.05). Except for hRV and hCoV, there was a significant difference in detecting the remaining viruses between age groups (*P* < 0.05) (Table 1).

**Table 1:** Detection of respiratory viruses in gender and age groups

	Gender			Age group				
	Male,No.(%)	Female,No.(%)	*P*-value	< 1,No.(%)	1–3,No.(%)	3–6,No.(%)	≥ 6,No.(%)	*P*-value
	*n* = 1040	*n* = 623		*n* = 982	*n* = 326	*n* = 233	*n* = 122	
IFV	36(3.5)	17(2.7)	0.41	21(2.1)	18(5.5)	10(4.3)	4(3.3)	0.017
RSV	146(14)	72(11.6)	0.147	162(16.5)	37(11.3)	14(6)	5(4.1)	< 0.001
hAdV	32(3.1)	16(2.6)	0.549	16(1.6)	13(4)	14(6)	5(4.1)	0.001
hPIV-3	81(7.8)	40(6.4)	0.299	88(9)	23(7.1)	6(2.6)	4(3.3)	0.002
hRV	149(14.3)	83(13.3)	0.567	125(12.7)	57(17.5)	33(14.2)	17(13.9)	0.202
hCoV	35(3.4)	21(3.4)	0.893	30(3.1)	14(4.3)	8(3.4)	4(3.3)	0.762

Chi-tests are the comparison of the number of respiratory virus detections between gender and age groups

**Fig. 1:**
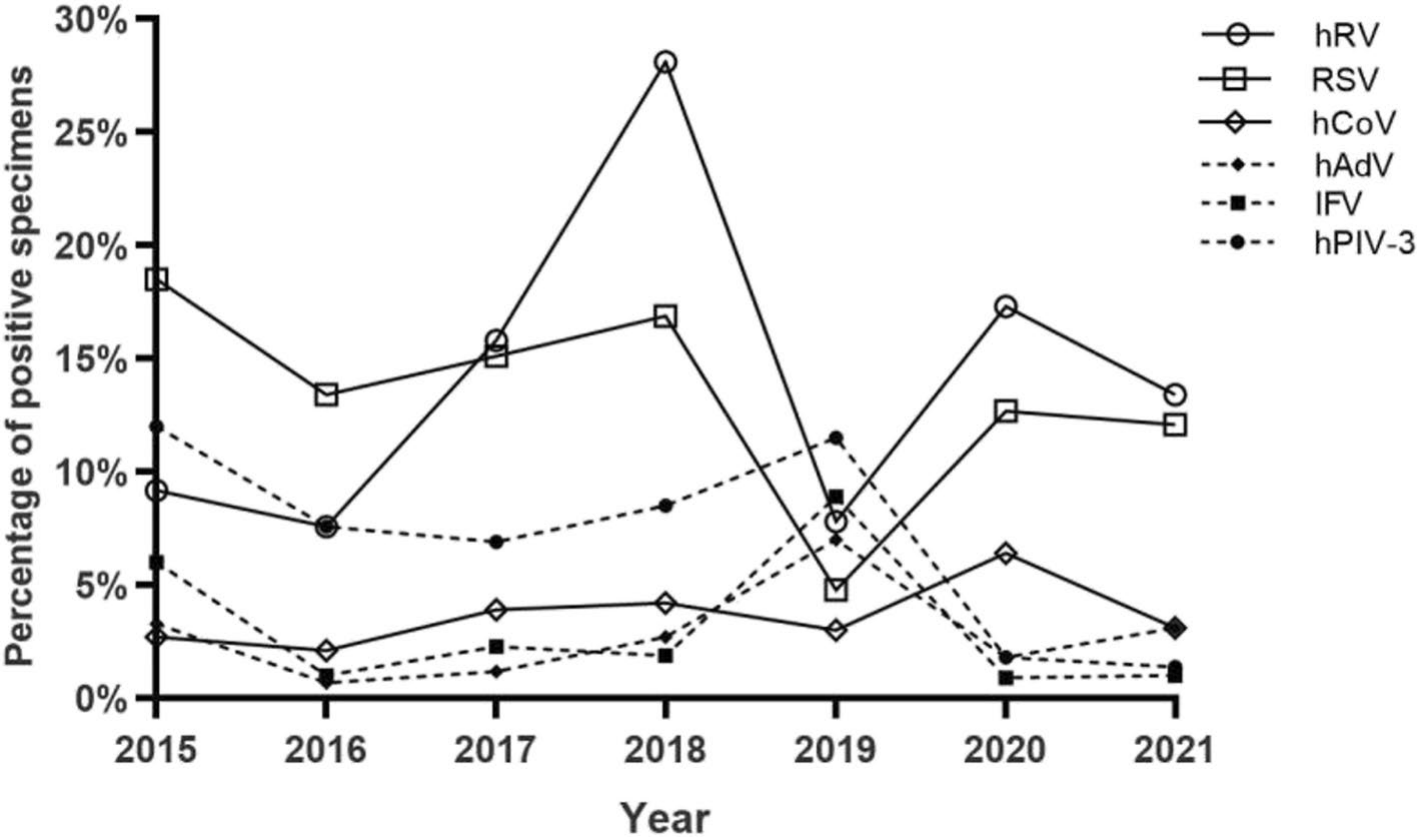
Changes in the detection rate of respiratory viruses, 2015–2021

The total detection rate of respiratory viruses was 38.5% (641/1663). The detection rates from high to low were hRV 14% (232/1663), RSV 13.1% (218/1663), hPIV-3 7.3% (121/1663), hCoV 3.4%, (56/1663) IFV 3.2% (53/1663) and hAdV 2.9% (48/1663). The detection rates of all viruses, except for hCoV, significantly differed between years (*P* < 0.01) (Table 2). Changes in viral detection rates are shown in Fig. 1.

**Table 2:** Respiratory virus detection in SARI cases, 2015–2021

	2015 No.(%)	2016 No.(%)	2017 No.(%)	2018 No.(%)	2019 No.(%)	2020 No.(%)	2021 No.(%)	Total No.(%)	*P*-value
	*n* = 184	*n* = 290	*n* = 259	*n* = 260	*n* = 270	*n* = 110	*n* = 290	*n* = 1663	
IFV	11(6)	3(1)	6(2.3)	5(1.9)	24(8.9)	1(0.9)	3(1)	53(3.2)	< 0.01
RSV	34(18.5)	39(13.4)	39(15.1)	44(16.9)	13(4.8)	14(12.7)	35(12.1)	218(13.1)	< 0.01
hADV	6(3.3)	2(0.7)	3(1.2)	7(2.7)	19(7.0)	2(1.8)	9(3.1)	48(2.9)	< 0.01
hPIV-3	22(12.0)	22(7.6)	18(6.9)	22(8.5)	31(11.5)	2(1.8)	4(1.4)	121(7.3)	< 0.01
hRV	17(9.2)	22(7.6)	41(15.8)	73(28.1)	21(7.8)	19(17.3)	39(13.4)	232(14)	< 0.01
hCoV	5(2.7)	6(2.1)	10(3.9)	11(4.2)	8(3)	7(6.4)	9(3.1)	56(3.4)	0.45

chi-tests are the comparison of respiratory viruses between years

### Comparison of detection rates of respiratory viruses before and after control measures

Before the control measures were taken, the total detection rate of respiratory viruses was 46.2% (2015–2019), and after the control measures, the detection rate decreased to 36% (2020–2021)(*P* < 0.01) (Table 3). Before taking control measures, the detection rates of IFV, hPIV-3, RSV and hAdV were 3.9%, 9.1%, 13.4% and 2.9%, respectively. After control measures, the detection rates of the four viruses were 1%, 1.5%, 12.3% and 2.8%, respectively (Table 3). The results showed that after the measures, the detection rate of four viruses was significantly lower than that before. However, the detection rates of hRV and hCoV were increased after control measures. Before the control measures, the detection rates of hRV and hCoV were 13.8% and 3.2%, respectively. After control measures, the detection rates of the two viruses were 14.5% and 4.1%, respectively.

**Table 3:** Comparison of detection rates of respiratory viruses before and after control measures

	Respiratory viruses						
	Total,No(%)	IFV,No(%)	hPIV-3,No(%)	RSV,No(%)	hRV,No(%)	hAdV,No(%)	hCoV,No(%)
2015–2019,*n* = 1263	584(46.2)	49(3.9)	115(9.1)	169(13.4)	174(13.8)	37(2.9)	40(3.2)
2020–2021,*n* = 400	144(36)	4(1)	6(1.5)	49(12.3)	58(14.5)	11(2.8)	16(4)
*P*-value	< 0.01	< 0.01	< 0.01	0.56	0.72	0.85	0.42

2015–2019 indicates before the control measures. 2020–2021 indicates after the control measures. Chi tests are the comparison between the number of positive detection in 2015–2019 and the number of virus positive detection in 2020–2021

**Fig. 2:**
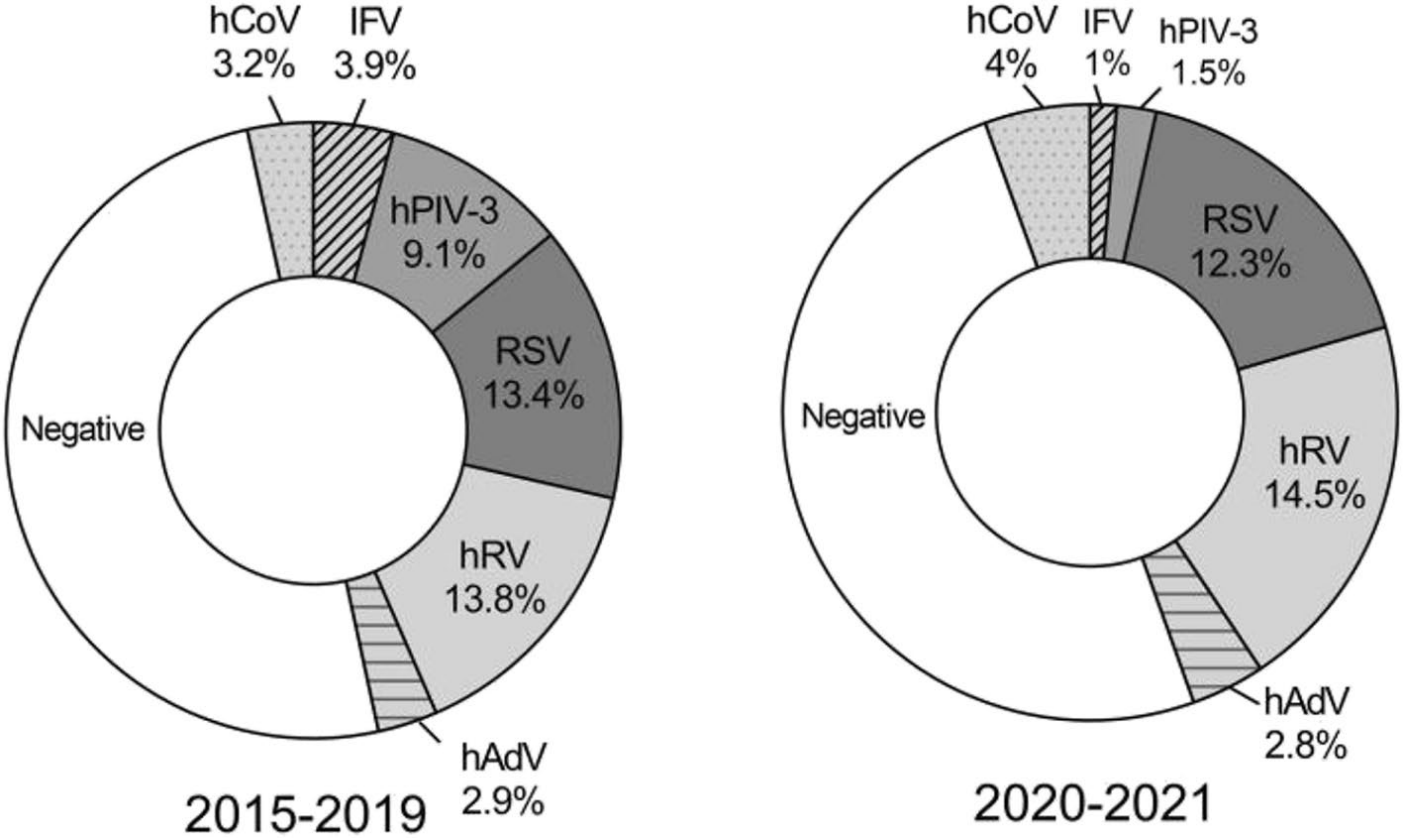
Changes in virus spectrum before and after the control measures

Before the control measures, the hRV, RSV, and hPIV-3 ranked in the top three, with detection rates of 13.8%, 13.4%, and 9.1%, respectively (Fig. 2). However, after the control measures, the detection rate of hPIV-3 decreased significantly(*P* < 0.01). hCoV replaced hPIV-3 to become the top three viruses in the detection rate. Finally, the three viruses with the highest detection rates were hRV, RSV, and hCoV, with 14.5%, 12.3%, and 4%, respectively.

### Monthly distribution of respiratory viruses before and after control measures

**Fig. 3:**
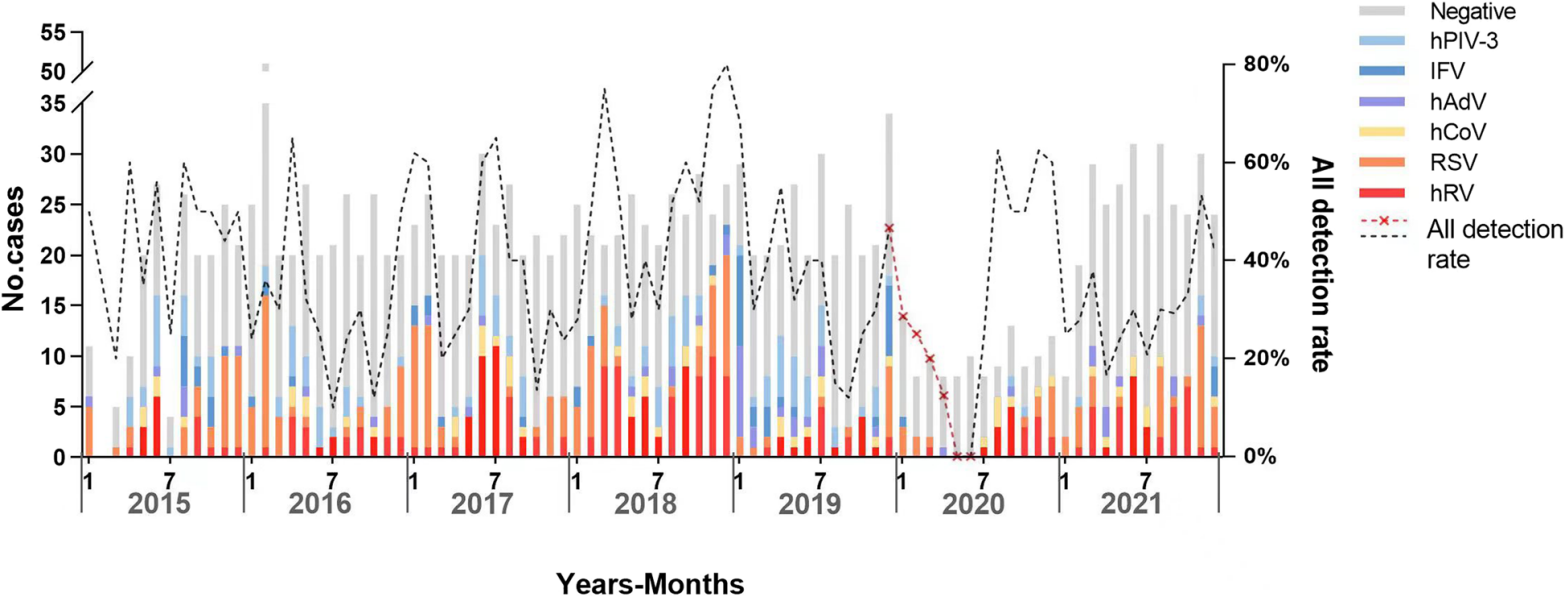
Monthly distribution of respiratory viruses from 2015–2021. The stacked bar chart corresponds to the left y-axis and indicates the number of positive and negative specimens for respiratory viruses for each month; the line graph corresponds to the right y-axis and shows the monthly detection rate of respiratory viruses

In 2020, the total detection rate of respiratory virus showed a V-shaped trend. From January to June 2020, the total virus detection rate continued to decline, from 28.6% in January to 0% in June. The average detection rate from January to June was lower than that of the same period from 2015 to 2019 (Fig. 3). Subsequently, the detection rate rose rapidly to 25% in July and steeply increased to 62.5% in August, and the average detection rate from July to December was higher than that of the same period from 2015 to 2019. However, the detection rate dropped again to 25% in January 2021 due to the impact of a new outbreak. In general, the detection rate in 2021 was lower than that in 2015–2019, although the detection rate was higher in March, October, and November.

**Fig. 4:**
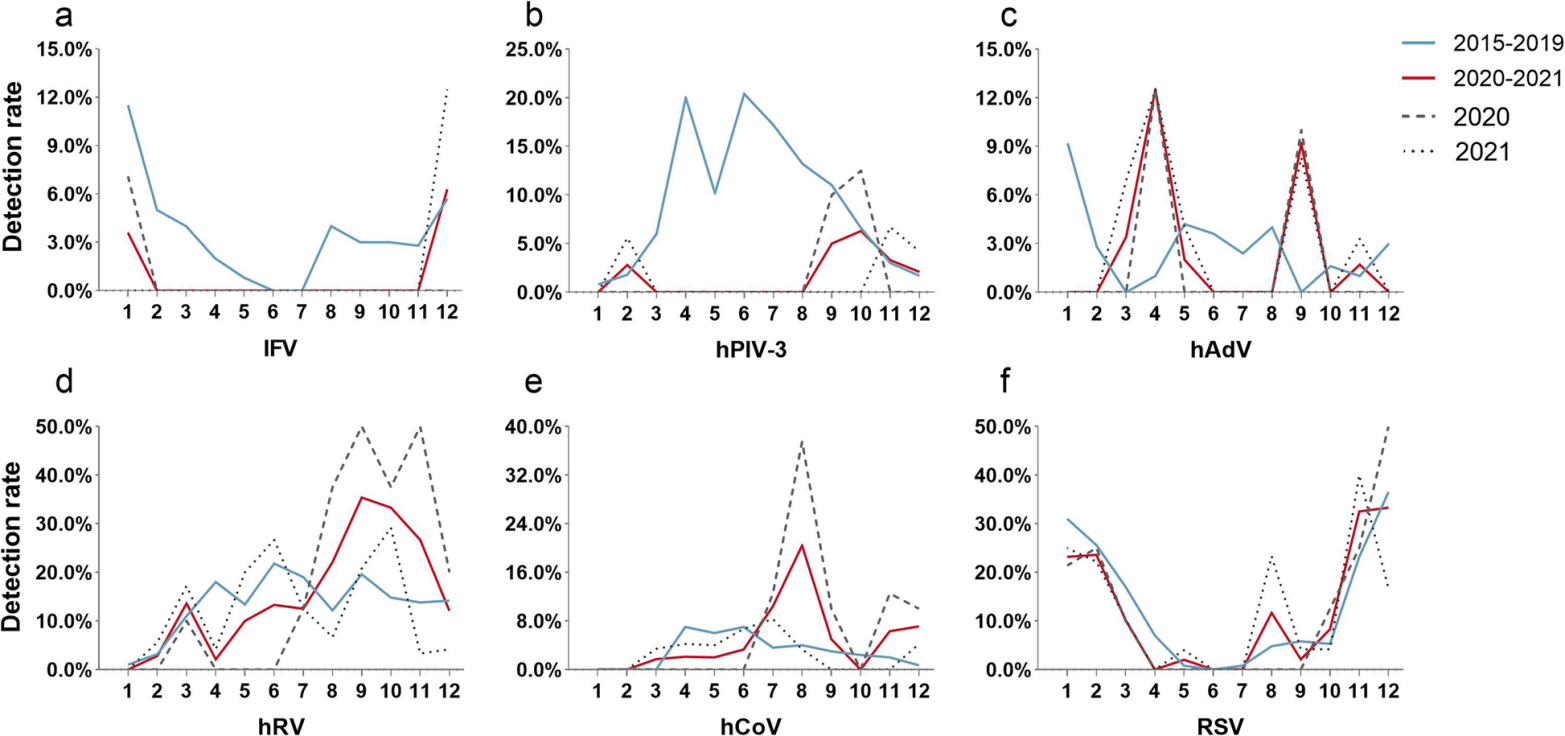
Comparison of the monthly distribution of each virus according to its detection rate in 2015–2021. The blue line represents the average monthly detection rate from 2015–2019, the red line represents the average monthly detection rate from 2020–2021, the dotted line represented the monthly detection rate in 2020, and the dotted line represented the monthly detection rate in 2021

From 2015 to 2019, the average detection rate of IFV decreased continuously from January to June, and then the monthly detection rate gradually increased from July. However, the prevalence of IFV decreased significantly after the control measures(*P* < 0.01). Surprisingly, IFV was not detected until December 2021 (Fig. 4a). The peak of hPIV-3 prevalence was between March and August before the control measures. However, after the control measures, the hPIV-3 could not be detected between March and August (Fig. 4b). From 2015–2019, the average detection rate of hAdV declined rapidly from January, the lowest in March, and then gradually increased. From April to August, the average detection rate of hAdV remained at about 3%, fell to the lowest again in September. However, the prevalance trends of hAdV reversed after the control measures. The peak months of hAdV prevalence were changed to April and September, with 12% and 9% detection rates, respectively (Fig. 4c). Although the detection rates of hRV and hCoV in August-December 2020 and July–September 2020, respectively, were higher than in the same period previously(*P* < 0.01), the overall prevalence trend did not change (Fig. 4d, e). The epidemic season of RSV was autumn and winter before and after the control measures, and no significant changes in monthly detection rates were observed (Fig. 4f).

### Change of respiratory virus spectrum in different age groups before and after control measures

**Fig. 5:**
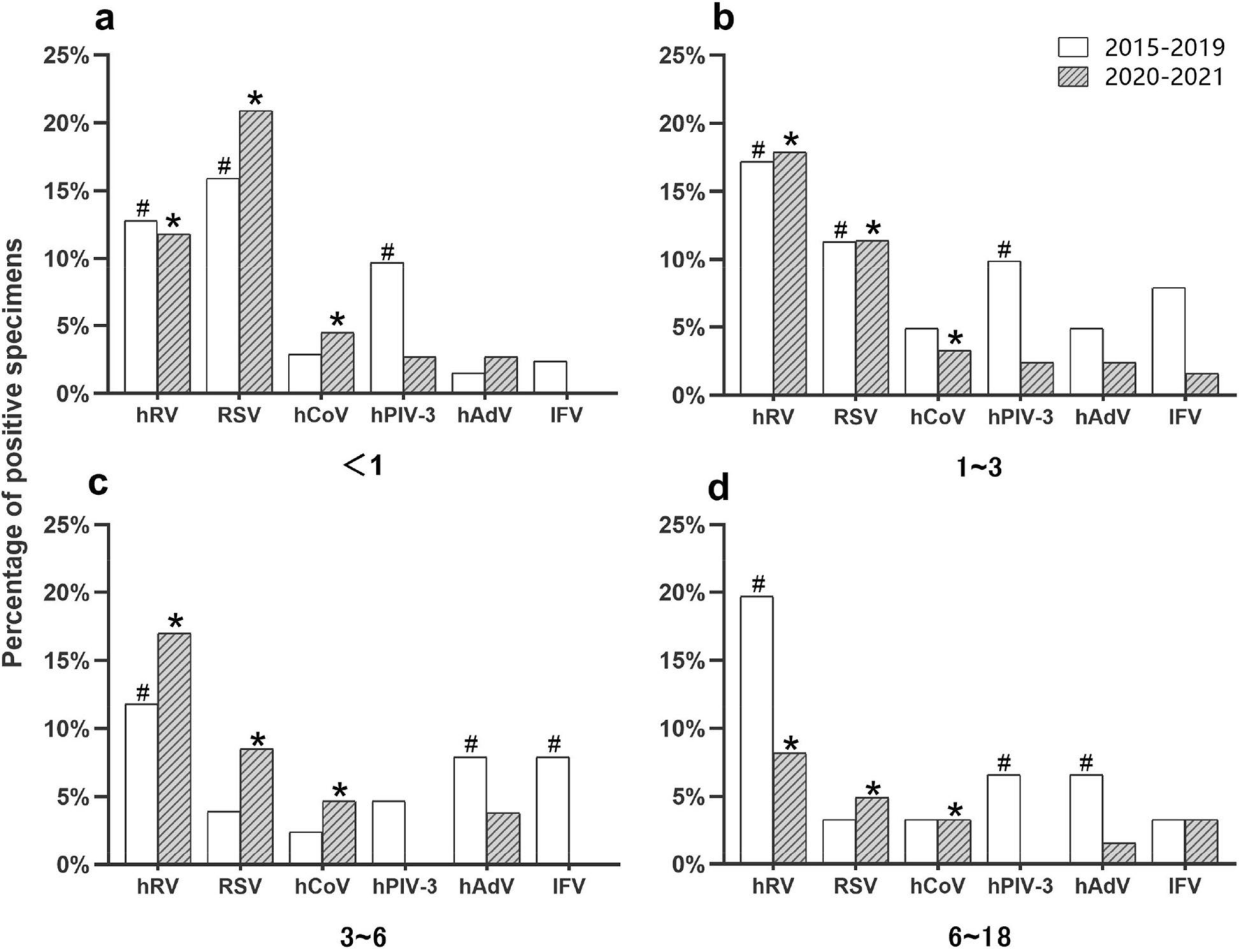
Change of respiratory virus spectrum in different age groups before and after control measures **#** indicates the top three viruses detected before the control measures, * indicates the top three viruses detected after the control measures

The inpatient children were divided into four groups according to their age (Fig. 5). The four groups were < 1 year old group, 1 ~ 3 years old group, 3 ~ 6 years old group, and 6 ~ 18 years old group, respectively. Before the control measures, hRV, RSV, and hPIV-3 ranked the top three in the detection of viruses in the < 1 year old and 1 ~ 3 years old groups. And hRV and hAdV were the top two in the detection of viruses between 3 and 18 years old. After the control measures, hRV, RSV, and hCoV were the top three in the detection of viruses in all age groups. The detection rate of hPIV-3 decreased in all age groups, while the ranking of the detection rate of hCoV increased in all age groups except the 1 ~ 3 years old group. No matter before or after the control measures, the RSV detection rate of children under 3 years old was at a high level. Among them, RSV was the virus with the highest detection rate in the < 1 year old group (Fig. 5a). As children grew older, hRV gradually became the most detected virus in the 1 ~ 3 years old group, 3 ~ 6 years old group, and 6 ~ 18 years old group (Fig. 5b ~ d).

## Discussion

Viruses are the primary pathogens of acute respiratory infections in children [17]. This study analyzed the detection of respiratory viruses in SARI cases among hospitalized children in Xuzhou from 2015–2021. The results showed that hRV, RSV, and hPIV-3 were the three viruses with the highest detection rates. The results of the Chinese respiratory infectious disease surveillance data showed that hRV and RSV are the main viruses causing pneumonia in children in recent years, with hPIVs ranking third and the detection rate exceeding IFV [18]. The control measures for the COVID-19 outbreak have curbed the spread of respiratory viruses among children(*P* < 0.01) The detection rates of hRV and RSV remained in the top two places before and after the control measures, indicating that the control policy may did not affect their prevalence in the region.

The Xuzhou city government has taken effective measures to prevent the spread of the epidemic since January 2020. Patients who test positive for Severe acute respiratory syndrome coronavirus 2 (SARS-CoV-2) are admitted to designated hospitals for quarantine and treatment. Therefore, the samples collected in this study were all SARS-CoV-2 negative samples. In July 2020, regular epidemic prevention and control measures were implemented, with public places reopening and public transportation resuming operations. However, the public was still required to wear masks when going out. Our results showed that the average detection rate from January to June was significantly lower than that of the same period from 2015–2019. However, after the regular control measures in July 2020, the average detection rate from July to December was higher than that of the same period from 2015–2019.The gradual resumption of offline teaching activities in early childhood institutions and primary and secondary schools from mid to late June after regulation of control measures, followed by the summer vacation with increased travel and the start of the school season in September. These may lead to the decrease of virus detection rate and then increase.

The implementation of control measures changes the prevalence of some viruses. Similar to the results of multi-country studies in the northern hemisphere [19–21], the reduction in the detection rate of IFV in this study was very significant (*P* < 0.01). The same results were found for countries in the southern hemisphere and tropical regions [22–24]. Surveillance data from the National Influenza Center in China shows an increasing trend in IFV activity levels until September 2021 with an absolute predominance of IFV-B [25]. Therefore, IFV is likely to resume epidemic after regulation of control measures [26, 27]. Several studies show that the epidemic season of hPIV-3 usually occurs in summer [28–30]. However, the trend of hPIV-3 reversed before and after measures in this study. Its epidemic season changed from the previous common spring and summer (March-September) to autumn and winter (*P* < 0.01). This result is similar to a study on hPIVs in Beijing in 2020, where the detection rate of hPIV-3 did not rise until October 2020, while the prevalence peak was from May to July in previous years [31]. The epidemiological trend of hAdV after control measures is diverse. The prevalence of hAdV in Korea did not change significantly after 2020 and was epidemic throughout the year [32]. In a pediatric study in the United Kingdom, the detection rate of hAdV is shown to increase significantly after 2020 [33]. However, in this study, the trends of hAdV before and after measures were reversed. Its detection rate peaked in April and September after the control measures, while the detection rate was lower or even 0% in the same period in previous years (*P* < 0.01). Some studies show that the hAdV-C type is predominant in the post-2020 epidemic and susceptible children [34]. Therefore, it is worth further studying whether a change of hAdV type has occurred in Xuzhou, China.

RSV is the leading cause of SARI in hospitalized children in the autumn and winter [35, 36]. After the COVID-19 outbreak in 2020, the detection rate of RSV in Europe decreased significantly, and its detection rate remained low even in the reports of 2021 [37]. With the downgrading of the control level, a rapid rebound in RSV detection rates was observed in the United States and Australia [38, 39]. However, the overall prevalence trend of RSV in this study was not significantly affected, and it continued to keep a high prevalence in autumn and winter. Moreover, RSV is usually considered the most common respiratory virus among hospitalized children under two years of age [40]. Still, this study found RSV infection was high in the infant and toddler group < 1 year of age, suggesting a higher threat of RSV in the region. Similarly, although the overall prevalence trend of hRV did not change in this study, its detection rate was higher in August-December 2020 than in the same period in previous years (*P* < 0.01). This elevation in the short term has likewise been reported in several domestic and international studies, such as the rapid increase in hRV detection rate in Beijing from 13.77% in June to 37.25% in August, significantly higher than the 22.51% in August 2017–2019 [41]. hRV detection rates pick up rapidly in some parts of the UK two weeks after the start of the school year [42]. Japanese scholars have a similar find that there is a significant increase in hRV detection rates among children under ten during the epidemic [43]. The other studies show that non-pharmacological interventions, such as environmental disinfection and wearing masks, have less impact on the transmission of non-enveloped viruses like hRV [44–46]. And there may be contact transmission of hRV [47, 48], contributing to the transient increase in hRV detection rates.

The age distribution of respiratory virus infections changed after the control measures. Consistent with the decrease in the total detection rate of hPIV-3 (*P* < 0.01), the detection rate of hPIV-3 decreased in all age groups, indicating that hPIV-3 was more significantly affected by the control measures. Moreover, the ranking of the hCoV detection rate increased in all age groups after the control measures, making it the third most detected virus. Similar to the results of a large-scale study in Beijing, the detection rate of hCoV increased from 1.74% in previous years to 3.29% in 2020, making it the second virus that detected the most among children [49]. Some studies have shown a biennial epidemiological trend of hCoV transmission in some areas of China [50]. A co-epidemic of common hCoV and SARS-CoV-2, especially after the regulation of control measures, would be a challenge for disease prevention and control. Therefore, we need to strengthen the surveillance of hCoV in the future.

Moreover, in addition to nonpharmaceutical interventions that alter the epidemiology of viruses, mutual interference between viruses is one of the hypotheses for epidemiological trends. Studies have shown that some viruses, such as IFV and hRV, can interfere with the infection of other viruses in the host or population [51, 52]. For example, the hRV epidemic in the autumn of 2009 seemed to delay or interrupt Europe's influenza A pandemic [53–55]. Meanwhile, the 2009 RSV pandemic worldwide was delayed by influenza [56, 57]. These suggest reciprocal patterns of positive or negative associations between respiratory viruses [58, 59].

Finally, a limitation of this paper is the number of hospital visits and hospitalized children in 2020 was reduced due to the impact of the epidemic, resulting in a significantly lower number of samples for 2020. Therefore this may hide the real numbers of patients presenting with SARI. In addition, this study did not include some viruses that also cause respiratory symptoms in children, such as hMPV and hBoV. Considering the low detection rate of the above viruses relative to the viruses selected for analysis in this paper [18], they were not included in our study. However, this is still an area we should additionally analyze in subsequent experiments.

## Conclusions

Implementing control measures for the COVID-19 epidemic has curbed the spread of respiratory viruses among children in Xuzhou, China. Although the preventive effect may be limited for some viruses that multiple routes can transmit, it suggests that we can learn from the experience of prevention and control of the COVID-19 outbreak to reduce the prevalence of respiratory viruses by increasing individual awareness of prevention through public education.

## Data Availability

All data generated or analyzed during this study are included in this published article.

## Abbreviations

COVID-19: Coronavirus disease 2019
SARS-CoV-2: Severe acute respiratory syndrome coronavirus 2
SARI: Severe acute respiratory infection
IFV: Influenza virus
IFV-A: Influenza virus A
IFV-B: Influenza virus B
RSV: Respiratory syncytial virus,
hAdV: Human adenovirus
hRV: Human rhinovirus
hPIV-3: Human parainfluenza virus type 3
hCoV: Human coronavirus
hCoV-229E: Human coronavirus-229E
hCoV-OC43: Human coronavirus OC43
